# Body Size and Geographic Range Do Not Explain Long Term Variation in Fish Populations: A Bayesian Phylogenetic Approach to Testing Assembly Processes in Stream Fish Assemblages

**DOI:** 10.1371/journal.pone.0093522

**Published:** 2014-04-01

**Authors:** Stephen J. Jacquemin, Jason C. Doll

**Affiliations:** 1 Department of Biological Sciences, Wright State University – Lake Campus, Celina, Ohio, United States of America; 2 Department of Biology, Ball State University, Muncie, Indiana, United States of America; National University of Mongolia, Mongolia

## Abstract

We combine evolutionary biology and community ecology to test whether two species traits, body size and geographic range, explain long term variation in local scale freshwater stream fish assemblages. Body size and geographic range are expected to influence several aspects of fish ecology, via relationships with niche breadth, dispersal, and abundance. These traits are expected to scale inversely with niche breadth or current abundance, and to scale directly with dispersal potential. However, their utility to explain long term temporal patterns in local scale abundance is not known. Comparative methods employing an existing molecular phylogeny were used to incorporate evolutionary relatedness in a test for covariation of body size and geographic range with long term (1983 – 2010) local scale population variation of fishes in West Fork White River (Indiana, USA). The Bayesian model incorporating phylogenetic uncertainty and correlated predictors indicated that neither body size nor geographic range explained significant variation in population fluctuations over a 28 year period. Phylogenetic signal data indicated that body size and geographic range were less similar among taxa than expected if trait evolution followed a purely random walk. We interpret this as evidence that local scale population variation may be influenced less by species-level traits such as body size or geographic range, and instead may be influenced more strongly by a taxon’s local scale habitat and biotic assemblages.

## Introduction

Attributing stream fish assemblage dynamics to random or deterministic factors is a long standing theme of community ecology [1], [2]. A current paradigm is that assemblages are highly organized by a variety of abiotic and biotic variables dictated by geographic and evolutionary scale [3], [4]. Specifically, local assemblage variation is linked to local scale factors such as predation [5], competition [6], habitat quality [7], and regional scale factors such as watershed land use type and history [8], stream size [9], and geologic history [10]. Unexplained assemblage variation is typically attributed to random noise or other untested mechanisms. Ultimately, however, assemblage patterns or characteristics are an emergent product of variation at the population level [11].

In addition to biotic and abiotic scale dependent factors, body size and geographic range are not necessarily independent of assemblage variation [12]. An inverse relationship between body size and abundance is expected as a function of energetic constraints [13] in both terrestrial [14] and aquatic [15] assemblages/ecosystems. Furthermore, macroecological studies have demonstrated a relationship between body size and geographic range [16], [17]. The expectation is that larger sized individuals are more capable of long range movements and thus, exhibit increased range sizes.

However, the utility of body size and geographic range as model predictors to describe long term population dynamics is understudied. Conceptually, small bodied species are expected to exhibit greater population variation as a result of higher intrinsic rates of increase *r* [17]. Similarly, species with larger geographic ranges are expected to be generalists for environmental niches [18] and more likely to exhibit stable populations.

However, there are complications with testing the relationship between population variation and traits such as body size and geographic range. Traits are not independently distributed across species, due to varying lengths of shared evolutionary history among related species. Thus, comparative analyses account for the expected covariance structure across species, based on hypothesized evolutionary relationships. Testing for phylogenetic signal (e.g. Blomberg’s *K* [19]) provides predictable patterns concordant with expected levels of evolutionary covariance (Brownian motion model of an evolutionary random walk), or alternatively, covariances may be lower (indicating more diverging paths, or convergence of unrelated species) or higher (indicating more conserved traits). Furthermore, interpretation of phylogenetic signal values can facilitate conclusions regarding broad evolutionary process of trait convergence or divergence [20].

A second complication involves quantitative issues associated with incorporating phylogeny into models which describe variation among taxa and the presence of collinearity among predictors. Body size and geographic range are correlated [12], yet, the relationship between these predictors and variation in abundance is of great interest [21], [22]. Incorporation of phylogeny into a model describing variation in abundance while accounting for collinearity among predictors is problematic with generalized least squares methods. However, Bayesian inference is an alternative statistical methodology which has been shown to result in more precise parameter estimates in phylogenetic models while accounting for collinearity among predictors [23], [24], [25].

The primary objective of this study was to test if body size and geographic range influence long term variation in local scale stream fish species abundance. Our secondary objective was to evaluate the phylogenetic signal of body size and geographic range associated with the stream fishes represented in our study. We hypothesized that body size and geographic range are negatively related to increased variation in long term population dynamics. We expected that taxa with small bodies and small geographic ranges would exhibit greater temporal variation in abundance as a result of energetic constraints, *r* vs. K selection mode, and small environmental niche.

## Materials and Methods

### Field collection

Fish were sampled yearly at six sites from 1983 to 2010 in the West Fork White River in East-Central Indiana (Indiana Department of Natural Resources Permit – JCD # 10-0098; see Table 1). Fish were collected following Simon and Dufour [26] and the Ohio Environmental Protection Agency for assessment of streams in the Eastern Corn Belt Plains ecoregion (Ohio Environmental Protection Agency (OEPA)) in accordance with American Fisheries Society guidelines for the safe and ethical use of fishes in research (http://fisheries.org/). Sampling was completed at normal pool water levels while turbidity was less than 40 Nephelometric Turbidity Units. All sites were sampled with a boat mounted Smith-Root model 5.0 GPP electrofisher with a 5000-watt generator. Sampling proceeded on a linear reach for a distance of 15 times the wetted width with a minimum distance of 500 m. Fish were collected using a 3 mm stretch mesh net and placed into a live well for processing. All fish (see Table 1) were identified to species using regional keys [27], counted, and released at the site. Voucher specimens curated at the Bureau of Water Quality, Muncie, Indiana were also used for species identification. All sites were sampled as part of the Bureau of Water Qualities long-term fisheries monitoring program in White River.

**Table 1:** Species included in analysis with descriptions of CV (long term population variation among sites), maximum body size (cm), and geographic range (km^2^).

Taxa	CV (min – max)	Maximum body size (cm)	Geographic range (km^2^)
*Moxostoma spp.*	0.40 – 1.10	78	1787287
*Hypentelium nigricans*	0.52 – 0.96	61	1581169
*Catostomus commersonii*	0.72 – 1.39	64	8850545
*Minytrema melanops*	0.57 – 1.04	50	1739172
*Carpiodes cyprinus*	0.71 – 1.73	66	2364892
*Carpiodes velifer*	3.26 – 4.80	50	1110876
*Cyprinus carpio*	0.66 – 1.24	120	8850545
*Notropis rubellus*	2.00 – 4.80	9	1697659
*Cyprinella whipplei*	1.96 – 3.31	16	497304
*Cyprinella spiloptera*	1.02 – 3.32	12	1516050
*Notropis stramineus*	1.39 – 4.80	8.1	3024895
*Notropis photogenis*	1.11 – 2.09	14	481459
*Pimephales notatus*	1.33 – 2.75	11	2784561
*Luxilus chrysocephalus*	0.70 – 2.94	18	1200793
*Lythrurus umbratilis*	1.59 – 4.80	8.6	1260469
*Ictalurus punctatus*	1.24 – 4.69	127	4899489
*Ameiurus natalis*	1.00 – 3.09	47	3623129
*Dorosoma cepedianum*	1.29 – 3.17	52	4559801
*Ambloplites rupestris*	0.40 – 0.86	43	2204814
*Pomoxis nigromaculatus*	1.00 – 2.11	49	3196197
*Pomoxis annularis*	1.15 – 1.84	53	2985169
*Micropterus salmoides*	0.60 – 1.28	97	3468067
*Micropterus dolomieu*	0.47 – 0.97	69	1817217
*Lepomis gibbosus*	3.16 – 4.80	40	1980638
*Lepomis microlophus*	2.09 – 4.69	25	1523148
*Lepomis megalotis*	0.71 – 0.90	24	2693156
*Lepomis cyanellus*	0.78 – 1.00	31	3849721
*Lepomis macrochirus*	0.64 – 1.19	41	3574355
*Percina caprodes*	0.82 – 1.29	18	3911619
*Percina maculata*	1.11 – 2.88	11	2035511
*Etheostoma nigrum*	2.06 – 4.81	7.2	3713135
*Etheostoma blennioides*	1.26 – 4.21	17	818767

### Data summary

**Figure 1:**
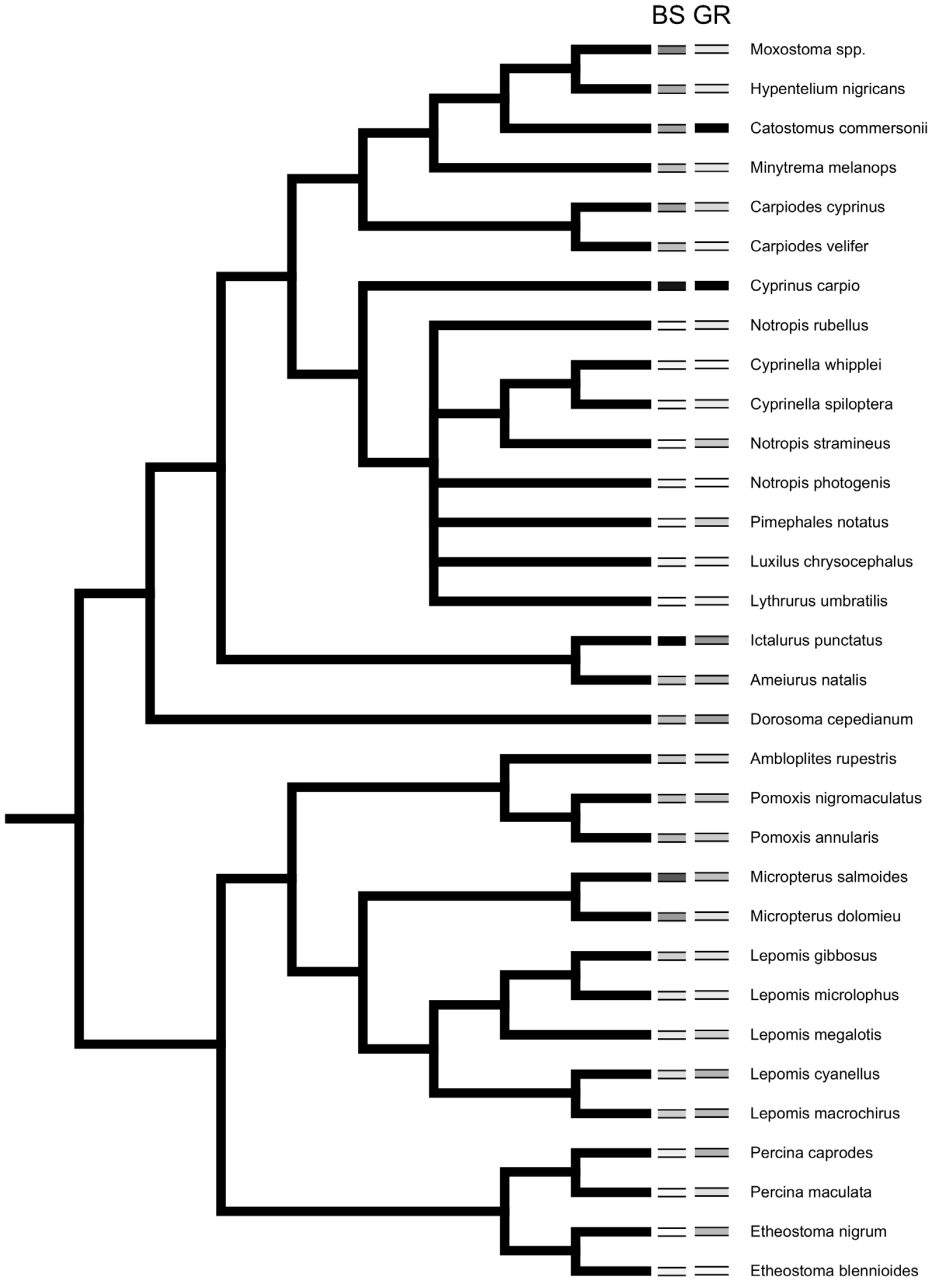
Phylogeny of study taxa with body size (BS) and geographic range (GR) categories. Darker bars indicate higher values. See Table 1 for raw data values.

Abundance per site was expressed as electrofishing catch per 1000 km. Body size and geographic range for each taxon were estimated using the Fish Traits database and standardized to their z-score (fishwild.vt.edu/fishtraits/[27]). Z-scores were calculated as follows: 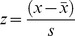
Where x is the observation, x^−^ is the mean value of the sample, and s is the standard deviation of the sample. The Fish Traits database has been concatenated from numerous regional and local distribution and life history studies [28] and can be used in large taxonomic scale studies [29]. Taxonomic relationships used in comparative analyses (Figure 1) were from published molecular studies of Catostomidae [30], Cyprinidae [31], [32], [33], Centrarchidae [34], Percidae [35], and Ictaluridae [36]. Higher order relationships (e.g. family) were from Betancur-R *et al.* [37].

### Statistical analysis

Long term variation in species abundance was estimated as the coefficient of variation, *cv*, for each species at each site. 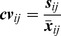
Where cv_ij_ is the coefficient of variation for species i at site j, s_ij_ is the standard deviation of species i at site j, and x^−^_ij_ is the mean abundance of species i at site j. Given the setup of the model only taxa that were collected at least once at each site over the collection period could be included. This resulted in a single species by site matrix of cv values (i.e. for species by sites).

**Figure 2:**
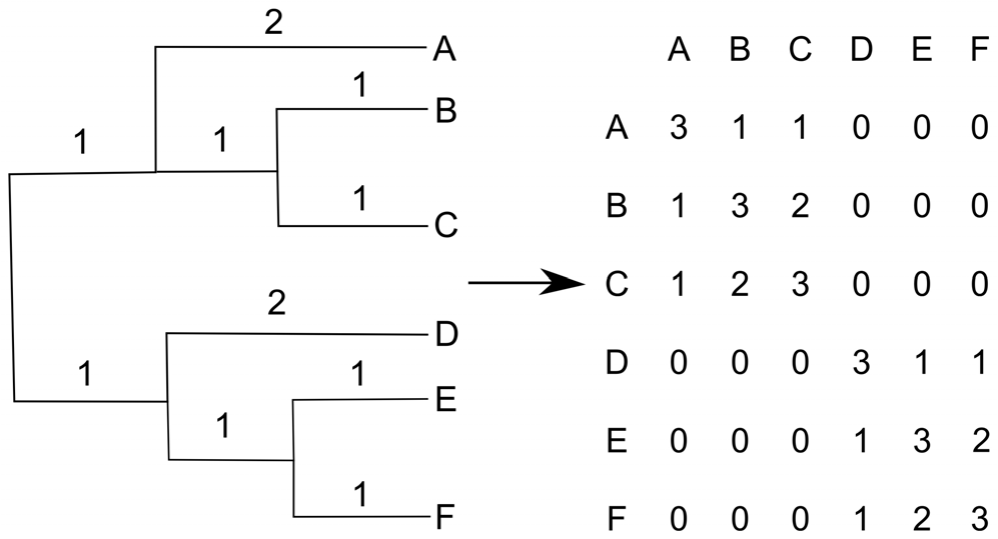
Variance-covariance matrix of a generalized phylogenetic tree. Variance is set to the branch length from the root to the tip and the covariance is the branch length from the root to the most recent common ancestor (adapted from de Villemereuil *et al.* 2012).



We modeled cv_ij_ as a linear function of body size and geographic range incorporating phylogenetic relationships following de Villemereuil *et al.* [23]. Here cv*_ij_* is modeled as a multivariate normal distribution where the mean is a linear function of body size, bs*_i_*, and geographic range, gr*_i_*, and the variance-covariance matrix, Σ, is proportional to the shared branch lengths from the root of the tree to the common ancestor of each pair of taxa (Figure 2).

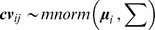


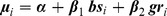
Where *μ_i_* is the mean of each species cv from the multivariate normal distribution (mnorm), α is the intercept and represents the hypothetical mean cv with a body size and geographic range of zero, and *β_1_* and *β_2_* are model coefficients representing the effect of body size and geographic range.

We used published molecular hypotheses to represent the phylogenetic relationship between species and used this single tree with an inverse-Wishart prior as a prior for the variance-covariance matrix, Σ [23]. We assumed equal branch lengths. As caveat to this analysis, if model parameters are identified as important the robustness of the model to choice of variance-covariance structure could be evaluated by generating and using a distribution of random trees [38] as the prior for the variance-covariance matrix.

Since body size and geographic range are known to be correlated [12] we used a Bayesian Lasso approach to include both variables in the model. The Bayesian Lasso is a variable selection technique that uses a double-exponential prior on the coefficients [24], [25]. The Bayesian Lasso will pull the weakest parameter to 0 thus providing a variable selection method with correlated predictors.

We used Bayesian inference to estimate parameters of the model. Bayesian inference is based on Bayes’ Theorem:
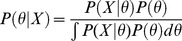



Where *P*(*X|θ*) is the likelihood function and represents the probability of the data, *X*, given the parameters, *θ*, *P*(*θ*) is the prior distribution of the parameters, *θ*, and the denominator is a normalizing parameter.

We used vague (i.e., noninformative) priors for all model parameters except the variance-covariance matrix, Σ, to specify our prior uncertainty about the model parameters. The variance-covariance matrix, Σ, prior was constructed as the inverse of the single phylogenetic tree matrix specified above in a Wishart prior. We used the freely available JAGS 3.3 program [39] implemented in R 2.15.3 [40] using the rjags package [41]. Complete model specifications in the JAGS language can be found in Appendix S1 of the Supporting Information. We ran 3 MCMC chains for a total of 125,000 steps, discarding the first 25,000 steps as a burn-in period, and thinning every 5 steps. The burn-in period is necessary to reduce the effect of the starting values on the MCMC results [42]. Convergence of the MCMC algorithm was assessed using the Brooks-Gelman-Rubin (BGR) scale-reduction factor [43]. The BGR factor is the ratio of between chain variability to within chain variability. Convergence is obtained when the upper limit of the BGR factor is close to 1.00 indicating there is not more variability between chains compared to within chains. Values below 1.10 are considered acceptable [42]. We additionally performed a posterior predictive check to evaluate model fit. This was conducted by calculating the posterior mean of the overall coefficient of variation for each species at each step in the Markov Chain. The 95% credible intervals from the estimated coefficient of variation was compared to the mean value for each species.

## Results

### Summary

The analysis included 48,071 individuals comprised of 32 species collected from 6 sites along the West Fork White River (Muncie, IN, USA) spanning 1983 to 2010 (Table 1). Taxon body size range was from 6.5 to 155 cm (mean 35 cm) and geographic ranges were from 481,459 to 8,850,545 km^2^ (mean 2,831,692 km^2^).

### Bayesian hierarchical model

**Figure 3:**
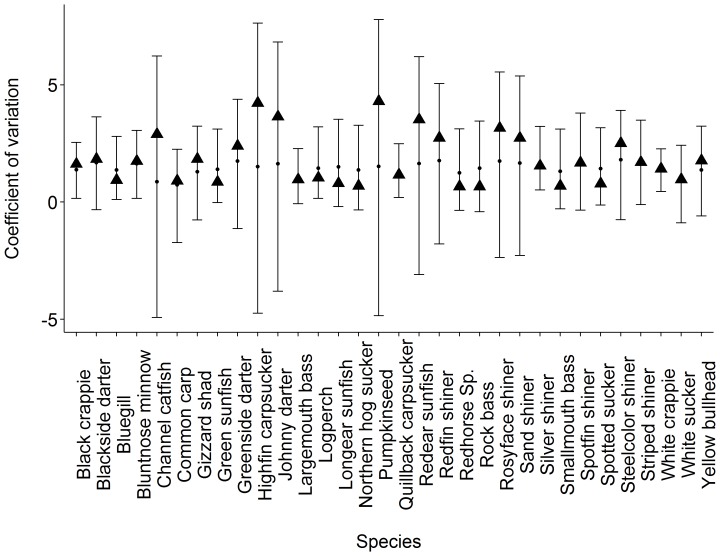
Results of the coefficient of variation posterior predictive check from the Bayesian hierarchical model. Points are mean estimates from the 95% credible intervals, vertical bars are the bounds of the 95% credible intervals, and solid triangles are observed mean coefficient of variation values for each species.

The BGR statistic for all parameters were less than 1.10 indicating the model converged after 100,000 iterations (33,333 steps per chain). The 95% credible interval estimates of the parameters for body size and geographic range overlapped 0 (Table 2), indicating there is no credible evidence to support a relationship with species coefficient of variation given the phylogenetic tree. When modeled separately with a normally distributed prior the posterior distribution of the body size and geographic range coefficient did not overlap 0. All of the 95% credible intervals from the posterior predictive check of the cv overlapped the observed mean value (Figure 3). Species with high observed average cv corresponded with a high credible interval values.

**Table 2:** Parameter estimates from the coefficient of variation model.

Effect	Median	95% Credible interval
Intercept	1.44	1.19 – 1.69
Geographic range	–0.08	–0.48 – 0.13
Body size	–0.17	–0.58 – 0.04

Parameter estimates are considered statistically significant when 95% credible intervals do not overlap 0.

### Phylogenetic signal

Body size exhibited low phylogenetic signal (*K* 0.57; *P<0.001;* Figure 1) indicating size distributions among taxa less similar than expected. Geographic range also had low phylogenetic signal (*K* 0.33; *P = 0.07;* Figure 1).

## Discussion

Long term variation in stream fish population abundances did not covary with body size or geographic range of taxa. This finding is contrary to our initial expectations; however, we do not interpret this as evidence that White River stream fish assemblages are random or stochastic. In a recent study of the same White River fish assemblage, Jacquemin and Doll [29] attributed a significant portion of the long term variation to differences in habitat and niche breadth (measured as association with particular substrate types, flow regime, woody debris, submerged vegetation, and distribution elevation) and responses to environmental variation among species. Specifically, Jacquemin and Doll [29] found that species with more general habitat niches showed smaller fluctuations in abundance through time. We interpret this as evidence that local scale stream fish assemblages are more closely aligned with environmental variation as a result of their respective niches than other traits such as body size or geographic range. However, while long term data provide a robust measure of local assemblage variation we suggest expanding spatial and taxonomic coverage through the addition of sites in other watersheds that may yield different results.

Ignoring multicollinearity in model parameters (e.g., body size and geographic range) can result in increased standard errors of the coefficients which can result in variables being found non-significant in traditional analysis. Thus, the relationship between variation in abundance with body size and geographic range is often conducted independently [16], [21], [22]. Typically, multicollinearity issues are addressed by increasing sample size or removing one of the intercorrelated variables. Increasing sample size is often not an option, particularly when analyzing long term data sets. Further, removal of a variable may not be an option when there is strong theoretical justification for including both. This study is the first to our knowledge that tests for a relationship between variation in abundance with body size and geographic range in the same model. The methods used here permit the inclusion of the correlated variables and provided a quantitative method of determining what variable is more important in driving variation in abundance when the correlated variables are considered important when tested individually.

Interestingly, our results for phylogenetic signal (low *K* values) of body size and geographic range implies less similarity among close relatives in the assemblage than expected under a Brownian model. Low *K* values are typically attributed to high levels of divergence, the opposite of niche conservatism [20]. One potential source of influence outside of divergence may also be our use of branch lengths in the analysis. Kraft *et al.* [44] interpret an assemblage level of highly ‘derived traits’ as evidence for habitat filtering influence on taxonomic assemblage variation. Further study of phylogenetic signal of ecologically relevant traits may improve understanding of assembly patterns in freshwater stream assemblages.

We suggest that our results are particularly relevant to conservation biology. Rabinowitz [45] and others [46], [47] identified utility in using life history traits to define rarity and extinction risk. Our results expand on these studies to indicate traits that may not covary with long term population dynamics. We suggest that while body size and geographic range did not contribute directly to long term variation at the population level that these species traits could explain variation at the assemblage level. Post hoc graphical observations of the dataset support a generally lower abundance among taxa that are larger and generally higher abundance among taxa that are smaller in the White River fish assemblage (as predicted in mammals [14], and for other North American fishes [16]). Ultimately, any information for long term covariates of threatened or endangered species could be incorporated into management plans. The inclusion of evolutionary relationships into community assembly studies can provide insight into species distribution patterns and population dynamics [48].

## Supporting Information

Appendix S1 (DOCX)
