# Supplementary material for: Body Size and Geographic Range Do Not Explain Long Term Variation in Fish Populations: A Bayesian Phylogenetic Approach to Testing Assembly Processes in Stream Fish Assemblages
Source: PLoS One. 2014 Apr 1;9(4):e93522. doi: 10.1371/journal.pone.0093522 (PMC3972097; doi:10.1371/journal.pone.0093522)
Supplement: Appendix S1 — (DOCX) [file pone.0093522.s001.docx]

Appendix 1.

model {

for (i in 1:sites){

spcv[i,1:S]~dmnorm(mu[],TAU[,])

}

for (j in 1:S) {

mu[j]<-alpha+beta[1]*range[j]+beta[2]*bs[j] # range = geographic range, bs = body size

}

for (k in 1:2){

beta[k] ~ ddexp(0, tau.beta) #prior for model coefficients

}

tau.beta<-lambda*tau.beta.2 #hyperprior for model coefficient

tau.beta.2~dgamma(0.001,0.001)

lambda~dgamma(0.001,0.001)

alpha~dnorm(0,0.0001)

TAU[1:S,1:S] ~ dwish(invA[,],S)

predicted[1:S]~dmnorm(mu[],TAU[,]) #Posterior predictive check
